# Data on the coefficient of static friction between surfaces coated with different sizes of rubber granules produced from used tires

**DOI:** 10.1016/j.dib.2019.01.022

**Published:** 2019-01-16

**Authors:** Ehab Hussein Bani-Hani, Jessica Lopez, Girish Mohanan

**Affiliations:** Mechanical Engineering Department, School of Engineering, Australian College of Kuwait, Mishref, Kuwait

## Abstract

The data on the static friction coefficient (*µ*) resulting between a surface and a friction block covered with shredded rubber produced from used commercial tires at varying granule sizes are presented. The tests are done using different types of plates such as, glass, Poly Vinyl Chloride (PVC) plastics, wood, concrete, marble, ceramic and sand paper to represent rough and smooth surfaces in contact with shredded rubber granule of sizes 1.18 mm, 0.6 mm, 0.425 mm, 0.3 mm, and 0.15 mm. The coefficient of static friction is calculated using Coulomb׳s Law of dry friction and the data are compared based on the granule sizes variability.

The data are represented in tables and figures. The data are in an almost uniform trend among the surfaces starting with a lower coefficient of friction for 1.18 mm to 0.6 mm, reached the highest at 0.425 mm and drops eventually as soon as the granules are finer.

**Specifications table**

**Table:** 

Tomasz Trzepieciński, Hirpa G. Lemu	T. Trzepieciński, H.G. Lemu, Study on frictional conditions of AA5251 aluminium alloy sheets using drawbead simulator test and numerical methods, Journal of Mechanical Engineering. 60 (2014) 51–60.

**Value of the data**

• The numerical values of the coefficient of static friction between the most used surfaces and the surfaces coated with rubber produced from used tires can be used in further relevant research directly with out the need to do experiments.
• The numerical values can be used in industrial sectors such as in concrete industry where crushed rubbers are used as aggregates in concrete manufacturing, fabricating the rubber surfaces in play areas, using recycled materials for environmental sustainability…etc.
• Some text books that explain the statics and dynamics systems need such data to give new examples about currently used materials. They need to explain static and dynamics friction through worked examples. Thus these data represent a good source of information.

## 1 Data

The data presented in this section are about the friction coefficient [1] between different surfaces that will be in contact with the rubberized surfaces. The coefficient of static friction data between ten widely used surfaces and surfaces coated with tires granules are measured experimentally [2] as shown in Table 1. Table 1 shows the average value of the coefficient of static friction (*µ*) for five crushed rubber sizes with standard deviation value (SD) for ten surfaces.

**Table 1:** The coefficient of friction for each rubber granule size at different surface plates.

**Collected data for frictional force**						
**Friction plate**	**Trial**	**Sieve no. 16**	**Sieve no. 30**	**Sieve no. 40**	**Sieve no. 50**	**Sieve no. 100**
		**Coefficient of friction, *μ***	**Coefficient of friction, *μ***	**Coefficient of friction, *μ***	**Coefficient of friction, *μ***	**Coefficient of friction, *μ***
Glass	1	0.38	0.38	0.48	0.29	0.31
	2	0.34	0.43	0.48	0.29	0.33
	3	0.38	0.43	0.48	0.29	0.34
	4	0.37	0.42	0.48	0.29	0.32
	***µ* ± SD**	**0.37 ± 0.02**	**0.42 ± 0.02**	**0.48 ± 0.00**	**0.29 ± 0.00**	**0.33 ± 0.01**
PVC	1	0.19	0.19	0.34	0.19	0.14
	2	0.14	0.19	0.29	0.19	0.15
	3	0.19	0.19	0.29	0.19	0.15
	4	0.18	0.19	0.30	0.19	0.15
	**µ ± SD**	**0.18 ± 0.02**	**0.19 ± 0.00**	**0.31 ± 0.02**	**0.19 ± 0.00**	**0.15 ± 0.00**
Ceramic	1	0.31	0.34	0.38	0.34	0.25
	2	0.29	0.29	0.38	0.36	0.25
	3	0.29	0.29	0.38	0.36	0.25
	4	0.30	0.30	0.38	0.35	0.25
	**µ ± SD**	**0.30 ± 0.01**	**0.31 ± 0.02**	**0.39 ± 0.00**	**0.36 ± 0.01**	**0.25 ± 0.00**
Marble	1	0.29	0.22	0.34	0.22	0.24
	2	0.24	0.22	0.38	0.22	0.22
	3	0.24	0.22	0.38	0.20	0.20
	4	0.26	0.22	0.37	0.21	0.22
	**µ ± SD**	**0.26 ± 0.02**	**0.22 ± 0.00**	**0.37 ± 0.02**	**0.21 ± 0.01**	**0.22 ± 0.02**
Wood Smooth	1	0.17	0.16	0.19	0.19	0.15
	2	0.19	0.16	0.19	0.19	0.14
	3	0.19	0.16	0.19	0.19	0.15
	4	0.19	0.16	0.19	0.19	0.15
	**µ ± SD**	**0.19 ± 0.01**	**0.16 ± 0.00**	**0.19 ± 0.00**	**0.19 ± 0.00**	**0.15 ± 0.00**
Concrete Smooth	1	0.34	0.38	0.43	0.38	0.36
	2	0.29	0.38	0.43	0.37	0.34
	3	0.34	0.38	0.43	0.39	0.33
	4	0.32	0.38	0.43	0.38	0.34
	**µ ± SD**	**0.34 ± 0.02**	**0.39 ± 0.00**	**0.44 ± 0.00**	**0.39 ± 0.01**	**0.35 ± 0.01**
Concrete Rough	1	0.62	0.58	0.67	0.60	0.58
	2	0.62	0.58	0.77	0.61	0.58
	3	0.67	0.58	0.67	0.61	0.59
	4	0.64	0.58	0.70	0.61	0.58
	**µ ± SD**	**0.64 ± 0.02**	**0.58 ± 0.00**	**0.71 ± 0.05**	**0.62 ± 0.00**	**0.59 ± 0.00**
Wood Rough	1	0.29	0.38	0.43	0.43	0.43
	2	0.38	0.38	0.43	0.44	0.43
	3	0.34	0.38	0.43	0.43	0.43
	4	0.34	0.38	0.43	0.44	0.43
	**µ ± SD**	**0.34 ± 0.04**	**0.39 ± 0.00**	**0.44 ± 0.00**	**0.44 ± 0.00**	**0.44 ± 0.00**
Sand Paper	1	0.77	0.82	0.86	0.58	0.58
	2	0.82	0.82	0.91	0.62	0.62
	3	0.75	0.86	0.91	0.67	0.58
	4	0.78	0.83	0.90	0.62	0.59
	**µ ± SD**	**0.78 ± 0.03**	**0.84 ± 0.02**	**0.90 ± 0.02**	**0.63 ± 0.04**	**0.60 ± 0.02**

**Fig. 1:**
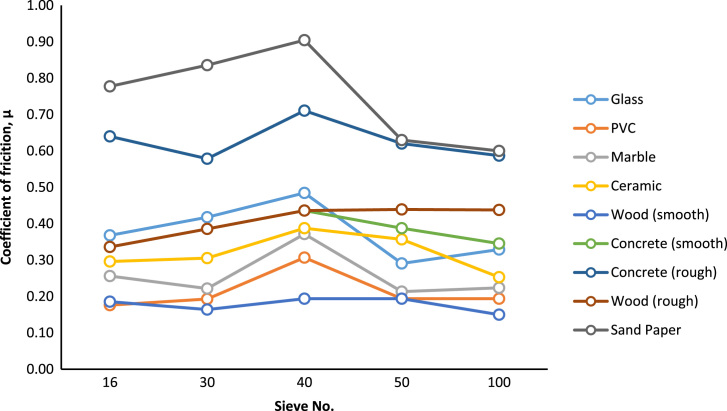
Coefficient of friction of various rubber granule sizes at different friction plate materials.

The general trend for each surface is shown in Fig. 1. This will provide very helpful data for the design, for example investigating the static equilibrium conditions and other applications.

## 2 Experimental design, materials and methods

In order to test the coefficient of static friction (*µ*) of surfaces coated with crushed rubber at different granule sizes, a commercial used tire was shredded. This shredded tire granules were sieved to five different sizes. After sieving, the collected granules were packed and labelled.

To gather sufficient data for comparison, these granules were tested with several different surfaces. A device that can measure static force and a constant sliding speed was utilized to create the friction between the granules and the surface. The coefficient of static friction was calculated for every reading and were compared with the rest of the granule sizes.

The procedure started with the preparation of the crushed rubber sample. The Endecotts EFL 2000 Sieve Shaker was used to sort the shredded rubber into different granule sizes. Sieve no. 16, 30, 40, 50 and 100 have a granule size of 1.18 mm, 0.6 mm, 0.425 mm, 0.3 mm and 0.15 mm respectively.

**Fig. 2:**
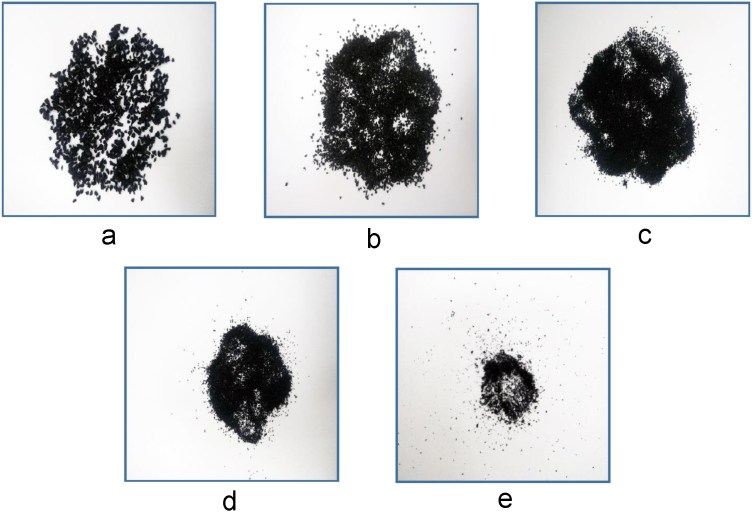
Shredded rubber granules collected after sieving. (a) Sieve no.16 (b) Sieve no.30 (c) Sieve no.40 (d) Sieve no.50 (e) Sieve no. 100.

Initially, the sieves were stacked on a reducing size order having one size bigger on top of sieve 16 and a collecting plate at the bottom. 300 g of shredded rubber were poured on the uppermost sieve and then the stack was placed on the sieve shaker. The machine ran for ten minutes and the gathered granules at each sieve were placed in separate containers. Fig. 2 shows the granules collected at each sieve plate.

**Fig. 3:**
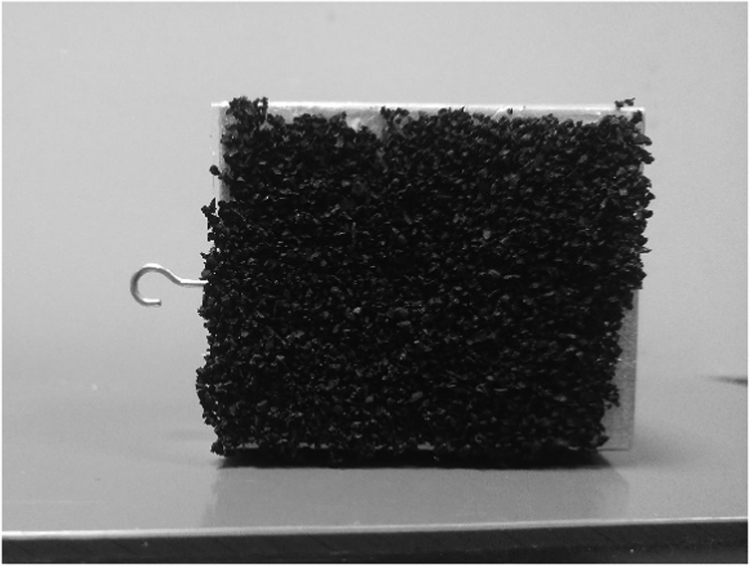
Friction block coated with shredded rubber granules.

This procedure was repeated for one kilogram of shredded rubber in order to have enough samples for each granule size. Then starting with biggest granule size, the shredded rubber were glued to cover one side of the surface of the friction block (Fig. 3) that weighs approximately one Newton. The new weight of the block with the rubber was recorded.

Using the G.U.N.T TM 120 Dry Friction Apparatus, the coefficient of friction between the rubber granules and a surface was determined by measuring the holding force from pulling the surface plate at a constant sliding speed. The friction plates used were Glass, PVC, Ceramic, Marble, Rough Wood, Smooth Wood, Rough Concrete, Smooth Concrete and Sand Paper. Each of the plates were placed on the carriage that was pulled by a 7.5 mm cable drum connected to a motor at a constant speed. The friction block was hooked to the string connected to the dynamometer measuring the holding force. The friction block was placed in the middle where the surface with the rubber coating is in contact with the friction plate. The motor was then turned on and as soon as the static friction was overcome, the holding force was recorded. This step was repeated three times and the average coefficient of friction was calculated. After this, the friction plate will be replaced with another one. The rubber granule size was changed after finishing all the tests for all the friction plates.

**Fig. 4:**
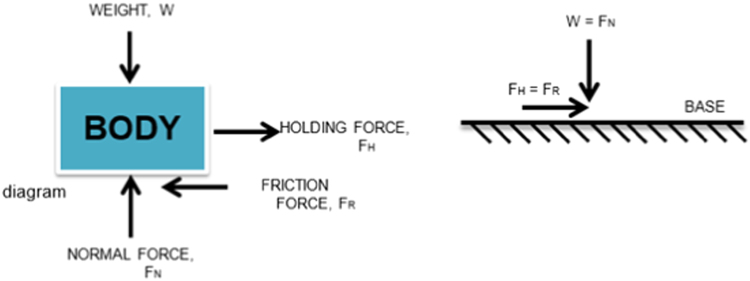
Free body diagram of the dry friction experiment.

Considering the free-body diagram (Fig. 4) of the experimental set-up, the normal force ($F_{N}$), is equal to the weight (W) of the friction block with the rubber granule coating and the frictional force ($F_{R}$) is equal to the holding force ($F_{H}$) given by the dynamometer.

To calculate the coefficient of friction, *μ*, the Coulomb׳s law Eq. (1) of dry friction was used given by the equation below;

(1) $$\mu \;=\;\frac{\mathrm{Frictional}\;\mathrm{Force}}{\mathrm{Normal}\;\mathrm{Force}}$$

The average value of the results from the three trials was calculated as shown in Eq. (2) and SD of the coefficient of friction for each granule size was calculated according to Eq. (3);

(2) $$\mathrm{Average}\;\mathrm{value}\;\mathrm{of}µ\;=\;\frac{\mu_{\mathrm{trial}\;1}+\mu_{\mathrm{trial}\;2}+\mu_{\mathrm{trial}\;3}+{µ}_{\mathrm{trial}\;4}}{4}$$

(3) $$\mathrm{SD}\;=\;\sqrt{\frac{\sum {\left({µ}_{i}-\mathrm{average}\;\mathrm{value}\;\mathrm{of}\;µ\right)}^{2}}{n-1}}$$

where

i is the trial number, 1, 2, 3, and 4
${µ}_{i}$ is the value of coefficient of static friction at trial i
n is the number of trials which is 4 trials
